# Heart Cells with Regenerative Potential from Pediatric Patients with End Stage Heart Failure: A Translatable Method to Enrich and Propagate

**DOI:** 10.1155/2012/452102

**Published:** 2012-08-14

**Authors:** Ann Steele, Robert J. Boucek, Jeffrey Phillip Jacobs, Peter Steele, Alfred Asante-Korang, Wilfredo Chamizo, Jasmine Steele, Paul J. Chai, James A. Quintessenza

**Affiliations:** ^1^The Congenital Heart Institute of Florida (CHIF), Saint Petersburg and Tampa, FL, USA; ^2^Seattle Children's Hospital Research Institute, University of Washington, 1900 Ninth Ave North, Seattle, WA 98101, USA; ^3^The Congenital Heart Institute of Florida (CHIF), All Children's Hospital, University of South Florida College of Medicine, Cardiac Surgical Associates of Florida (CSAoF), Saint Petersburg and Tampa, FL, USA; ^4^Department of Pathology and Laboratory Medicine, All Children's Hospital, Saint Petersburg, FL, USA; ^5^The Congenital Heart Institute of Florida (CHIF), All Children's Hospital, University of South Florida College of Medicine, Pediatric Cardiology Associates/Pediatrix, Saint Petersburg and Tampa, FL, USA; ^6^Nova Southeastern University, Ft. Lauderdale, FL, USA

## Abstract

*Background*. Human cardiac-derived progenitor cells (hCPCs) have shown promise in treating heart failure (HF) in adults. The purpose of this study was to describe derivation of hCPCs from pediatric patients with end-stage HF. *Methods*. At surgery, discarded right atrial tissues (hAA) were obtained from HF patients (*n* = 25; hAA-CHF). Minced tissues were suspended in complete (serum-containing) DMEM. Cells were selected for their tissue migration and expression of stem cell factor receptor (hc-kit). Characterization of hc-kit^positive^ cells included immunohistochemical screening with a panel of monoclonal antibodies. *Results*. Cells, including phase-bright cells identified as hc-kit^positive^, spontaneously emigrated from hAA-CHF in suspended explant cultures (SEC) after Day 7. When cocultured with tissue, emigrated hc-kit^positive^ cells proliferated, first as loosely attached clones and later as multicellular clusters. At Day 21*~*5% of cells were hc-kit^positive^. Between Days 14 and 28 hc-kit^positive^ cells exhibited mesodermal commitment (GATA-4^positive^ and NKX2.5^positive^); then after Day 28 cardiac lineages (flk-1^positive^, smooth muscle actin^positive^, troponin-I^positive^, and myosin light chain^positive^). *Conclusions*. C-kit^positive^ hCPCs can be derived from atrial tissue of pediatric patients with end-stage HF. SEC is a novel culture method for derivation of migratory hc-kit^positive^ cells that favors clinical translation by reducing the need for exogenously added factors to expand hCPCs *in vitro*.

## 1. Introduction

In the current era, the human heart is thought to possess some regenerative potential. Experimental evidence based on genetic fate mapping confirms that cardiac-derived stem or precursor cells (CPCs) contribute to replacement of adult mammalian cardiomyocytes [1]. These innate CPCs possess properties of stem cells, specifically, they are clonogenic, self-renewing, capable of asymmetric division, express stem cell markers (i.e., stem cell antigen receptor or c-kit), and are capable of differentiating into cells of the cardiac lineages, including endothelial cells, smooth muscle cells, conduction cells, and cardiomyocytes [2].

Heart failure is a disabling, costly, and potentially fatal disorder not only in adults but also in children. HF in pediatric patients is predominantly secondary to congenital heart disease (CHD) [3]. Surgical repair of CHD in children has been highly successful with low operative mortalities [4], normal hemodynamic results, and postoperative quality of life [5]. But heart failure (HF) can be a late complication of CHD in as many as 40% of adult-aged with CHD [6] and is a frequent indication for heart transplantation in children [7]. In the pediatric age group, CHD and cardiomyopathies are the predominate causes of heart failure leading to heart transplantation [8], with far fewer due to ischemic heart disease when compared with the adult age group [8]. Unfortunately, since most children have undergone surgical repairs for their CHD, they frequently have high levels of antibodies to HLA antigens that increases their risk of heart transplantation [9, 10]. For many pediatric patients with heart failure, an autologous cell-based regenerative strategy would be a better therapeutic option than allogeneic heart transplantation [11].

Pediatric HF is indicative of limited innate cardiac regeneration, possibly because cell losses exceed the limited regenerative potential of low numbers of innate hCPCs [11, 12]. Bone-marrow-derived cell therapy trials of adult patients [13] and case reports of pediatric patients [14, 15] with end-stage HF have shown improvement in heart function. In the absence of conclusive evidence for significant cardiomyocyte differentiation of these administered cells, the postulated mechanism for improved function has been a paracrine factor-mediated recruitment of innate CPCs [13, 16].

The mammalian heart contains cardiac stem or progenitor cells (CPC) that express the surface receptor tyrosine kinase c-kit. C-kit^positive^ cells were identified in human tissues initially by Anversa's group as cardiac-derived progenitor cells (CPC) [17]. It is now generally accepted that c-kit^positive^ cells can be identified in pediatric [18] as well as adult hearts [19]. Several groups have reported that c-kit^positive^ cells derived from cardiac tissue can differentiate *in vitro* to beating cardiomyocytes [20]. We reported that c-kit^positive^ cells derived from the hearts of syngeneic green fluorescent protein expressing mice, expanded by SEC, and administered to mice with coronary injury due to APO-E deficiency, localize to the vessel wall and differentiate to endothelial cells [21]. Following *in vitro* expansion, there have been numerous reports of benefits when c-kit^positive^ CPCs are administered to injury models. Early evidence from an ongoing clinical trial in adult patients with chronic HF has demonstrated improvements in heart function after administration of *in vitro *expanded autologous cardiac-derived hc-kit^positive^ cells [22].

These considerations have focused our interests on the *in vitro* expansion of hCPCs for autologous cell-based therapies for pediatric patients with CHD and end-stage HF. The methods described in literature to date rely on enzymatic disruption of tissues and supplementation of the media with growth factors [22]. With regulatory constraints in mind, existing methods for derivation of hc-kit^positive^ cells potentially create problems associated with the use of enzymatic digestion and culture supplements which can introduce non-autologous proteins, potential antigens, and risk for xenogeneic infections as well as altered cell surface protein markers that are necessary for precise cell characterizations [18, 23]. We have previously reported that suspension explant culture (SEC) of murine heart tissues facilitates the nondestructive derivation of c-kit^positive^ cells and sustains the *in vitro* expansion of c-kit^positive^ cells [21]. The aim of this paper is to describe the application of SEC to human heart tissues to promote the selection and expansion of hc-kit^positive^ cells with stem cell properties.

## 2. Methods

### 2.1. Patients

This study was approved by All Children's Hospital Institutional Review Board (IRB number 07-0028). Between November 2007 and November 2010, 25 pediatric patients undergoing surgery for CHD or orthotopic heart transplantation for end-stage heart failure from congenital or acquired heart disease were included in this study. All patients were in the pediatric age range; median age for the group was 4.6 years (range: 4 days to 16.5 years). For 13 patients, selected to span the pediatric age range of our patients (8 days to 16 years), the timing and reproducibility of cell emigration from heart tissues using SEC were determined. Characterization of the c-kit^positive^ population was performed on cells derived from all patients (*n* = 25) using a panel of monoclonal antibodies. Lastly, right atrial appendage tissue from three patients was used to determine yields and enrichment of c-kit^positive^ cells over time. 

### 2.2. Tissue Explant Culture

The emigration of cells from explanted cardiac tissue in culture has been previously noted [24]. We also recognized that some of these emigrating cells expressed stem cell markers [21]. The explant culture method used in this report was adapted for patient-derived, discarded heart tissues from previously reported culture methods for murine heart tissues [21]. This method, identified herein as suspension explant culture (SEC), is based on modifications of a method originally described by Messina et al. [24] and later modified by Smith et al. [23]. Using sterile technique, discarded human right atrial appendages were collected, weighed, and minced. The minced tissues were placed in high glucose (4500 mg/L) Dulbecco's Minimal Essential Media (DMEM; Invitrogen, USA) with added 20% fetal calf serum (FCS; InVitrogen, USA) and antibiotics (penicillin and streptomycin; InVitrogen, USA). To a T-75 Polypropylene culture flask (Corning, USA), 50 mls of complete media were added. This higher volume is an important modification to ensure that the tissue remains suspended and does not become adherent to the surfaces of the flask. All cultures were maintained in a humidified chamber at 37°C in 95% air/5% CO_2_. Every three days, 3 mL of the DMEM + FCS were removed and an equal volume of fresh complete media was added. Care was taken not to disrupt the cultures during handling. To this end, the refeeding was limited to ~6%v/v exchange of media. The rationale for this feeding strategy was to preserve any “conditioning” provided by putative paracrine factors derived from the retained cocultured tissues while resupplying nutrients. Cultures were examined daily using an inverted microscope (Leica, USA).

### 2.3. Processing and Characterization of Explant and Emigrated Cells

For cell counting or characterization, aliquots of loosely adherent emigrated cells were simply aspirated on collection without the use of enzymatic digestion. Adherent cells were harvested following brief (less than 30 seconds) enzymatic treatment (0.2%/1 mM trypsin/EDTA; InVitrogen, USA). All harvested cells were immediately fixed in suspension in 10% Neutral Buffered Formalin (NBF) to cross-link and minimize loss of critical surface membrane epitopes. In addition, fixation also stabilized the highly fragile, multicellular cardiospheres and enabled retention of the spatial relationships between cells within these structures. Fixed individual cell harvests and multicellular cardiosphere samples were prepared as monolayers on slides by centrifugation (1000 rpm@5 min, Shandon Cytospin, Thermo Scientific, USA).

Immunohistological characterization of tissue-derived single cells and of multicellular cardiospheres were performed as previously described [21]. For the purpose of this study, c-kit^positive^ cells were considered to be representative of CPCs in pediatric heart and similar to the cardiosphere-derived cells from adult human heart [18, 23]. Characterization panels included monoclonal antibodies (Vantana, USA or Santa Cruz Biotechnologies, USA) to stem cell markers (c-kit or CD117, Sca-1, Isl-1) transcription factors (GATA-4 and Nkx2-5), and differentiation/lineage markers(Flk-1, smooth muscle actin, troponin I, cardiac myosin light chain 2, muscle-specific actin, CD45, and CD34). Heart tissues after SEC were also fixed in 10% NBF and processed to paraffin for preparation of tissue sections and hematoxylin and eosin (H&E), trichrome, or immunohistochemical staining. Proliferating cells were identified in both fixed tissue and cellular cytospin preparations by immunohistochemical staining using proliferating cell nuclear antigen (PCNA; Santa Cruz Biotechnology, USA) [25]; only nuclear staining (metaphase) was considered positive. Antibodies to CD34 and CD45 (Vantana, USA) were used as internal negative controls. Control tissues included tumors, normal cardiac tissue, smooth muscle and bone marrow. Reporter chromogen 3, 3′ Diaminobenzidine (DAB) enabled visualization of bound antibody. Cell and tissue immunopreps were counterstained with hematoxylin.

### 2.4. CPC Yield from SEC

To estimate the number of hc-kit^positive^ cells derived from SEC and *in vitro* hc-kit^positive^ cell proliferation, triplicate subsamples (~0.1 g) of cardiac tissue from each patient (*N* = 3) were placed into individual T-25 polypropylene culture flasks (Corning, USA) containing 30 mL complete DMEM. Ten percent of each culture's media was exchanged for fresh media every 3 days. Cultures were harvested at Days 7, 14, 21, and Day 28. For counting purposes, nonadherent cells were collected using gentle aspiration and adherent cells were harvested with very brief trypsinization.

C-kit^positive^ cells were immunomagnetically selected as previously reported [26] using monoclonal antibody with high affinity for c-kit attached to iron-containing nanoparticles (EasySep kits #18757; Stem Cell Technologies, CA). Selected (c-kit^positive^) and nonselected (c-kit^negative^) cells were manually counted using a hemocytometer. Total c-kit^positive^ cell numbers were determined for each sample time point and for measured tissue wet weights.

## 3. Results

Discarded right atrial appendages were collected and, when possible, weighed under sterile conditions prior to SEC. The median wet weight was 1.19 g (*n* = 25; range: 0.21 to 10.9 g).

**Figure 1:**
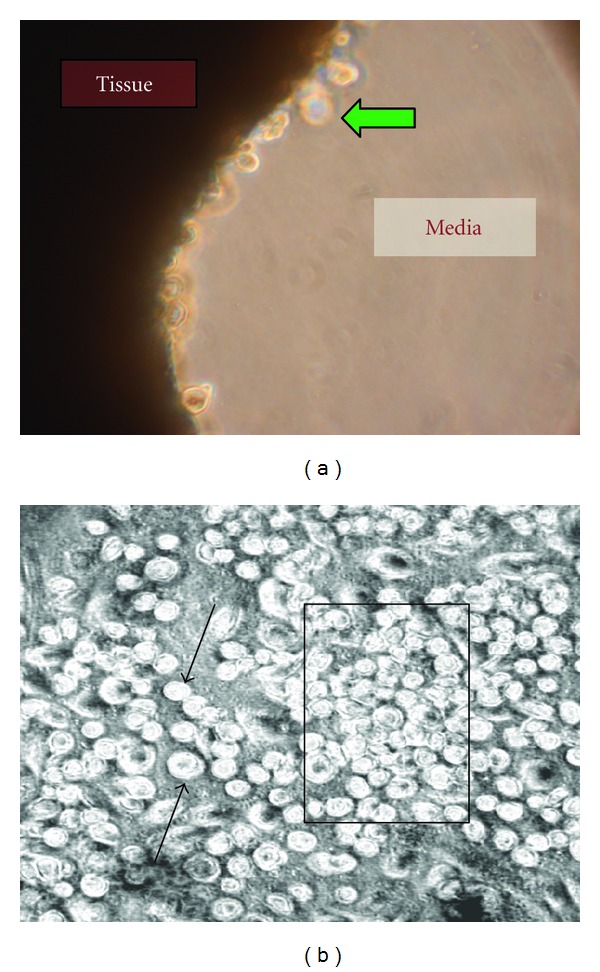
Representative phase contrast photomicrographs of cell emigration and proliferation with SEC. (a) Spontaneous emigration of phase-bright cells (green arrow) “budding” or emerging from the cut surface of suspended atrial tissues at Day 7 of SEC. Magnification ×400. (b) Loosely attached phase-bright single cells (arrows) and cellular aggregates (box) at Day 21 in SEC. Magnification ×250.

**Figure 2:**
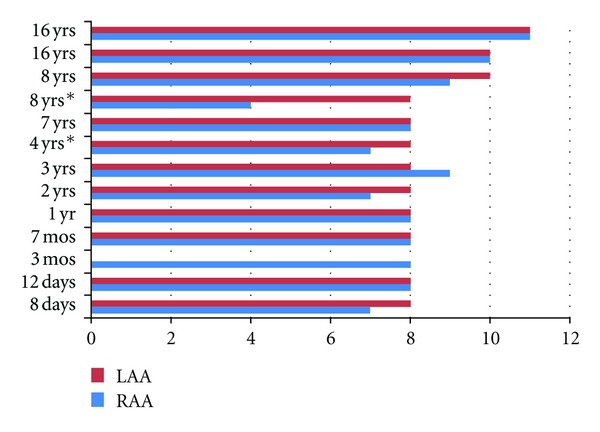
Time-to-first emigration. The time in days to first phase-bright cell emigration is displayed for a subgroup with right arterial appendage (RAA) and left atrial appendage (LAA) in a group of patients selected to span the pediatric age range of our patients (8 days to 16 years). All of the patients, except the two patients marked with an asterix, had end-stage heart failure at the time that the atrial appendages were removed.

With SEC, spontaneous emigration of phase-bright, mononuclear cells from the suspended explanted cardiac tissue was observed from cultured atrial tissues from all 25 patients with chronic heart failure (Figure 1(a)). Emigrated phase-bright cells loosely attached to the bottom of the flask, initially as single cells (arrows) and by Day 21 appear as tight aggregates suggesting clonal expansion (Figure 1(b); rectangular box). With SEC there was a stereotypic time-dependent emigration and expansion of cardiac-derived hc-kit^positive^ cells observed for the 25 patient samples regardless of patient age or diagnosis. The initial appearance of emigrated cells from right compared to left atrial appendage for 13 pediatric patients selected to span the pediatric age range of our patients (8 days to 16 years) is shown in Figure 2.

Emigrated phase bright cells were first observed as early as Day 4 and as late as Day 11 (Figure 2). The average time-to-first emigration for RAA and LAA tissues were not different (8 versus 8.6 days; *n* = 13). However, there was a trend to longer time to emigration with age of the patient (*p* = NS).

**Figure 3:**
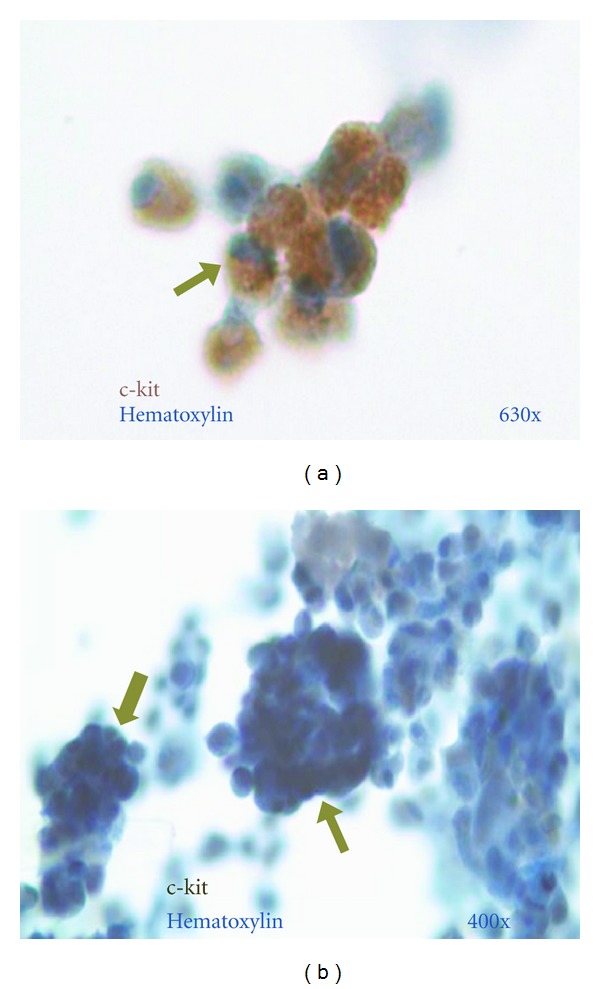
Identification of emigrated cells as c-kit^positive^. (a) Cluster of c-kit^positive^ cells from a cytospin preparation after 28 days in culture (c-kit^positive^ cells (light- and dark-brown arrow) visualized using reporter chromogen DAB with nuclear hematoxylin counterstain (light blue). Magnification ×630. (b) Representative cardiosphere composed of multiple cells (arrows) that were predominantly c-kit^positive^ (brown to black-nickel-toned DAB and light blue nuclear hematoxylin counterstain). Magnification ×400.

Some of the cells that first emigrated expressed c-kit (Figure 3(a)). Between Day 10–14, a small subpopulation of c-kit^positive^ cells spontaneously aggregated to form spheroid structures (Figure 3(b)) called cardiospheres [24]. Cardiospheres were composed initially of c-kit^positive^ cells (Figure 3(b)) and were interspersed among the larger population of attached c-kit^positive^ single cells. At first, cardiospheres were small (~30 *μ*m) but by Day 28 the number of cells comprising the spheres increased forming large cardiospheres that were >150 um in diameter. Cardiospheres are analogous to the embryoid bodies formed by embryonic stem cells [27] and, as previously reported, represent a clonal expansion of the cardiac-derived c-kit^positive^ cells [24].  Figure 3 is a representative photomicrograph of c-kit expression by cells (Figure 3(a)) and cardiospheres (Figure 3(b)) after 28 days in SEC. C-kit expression was noted for both individually occurring cells and for multicellular SEC-derived cardiospheres for all 25 patients. We have previously reported durable c-kit^positive^ expression using flow cytometry analyses of cells derived by SEC culturing from murine heart at 12 weeks in culture [21].

**Figure 4:**
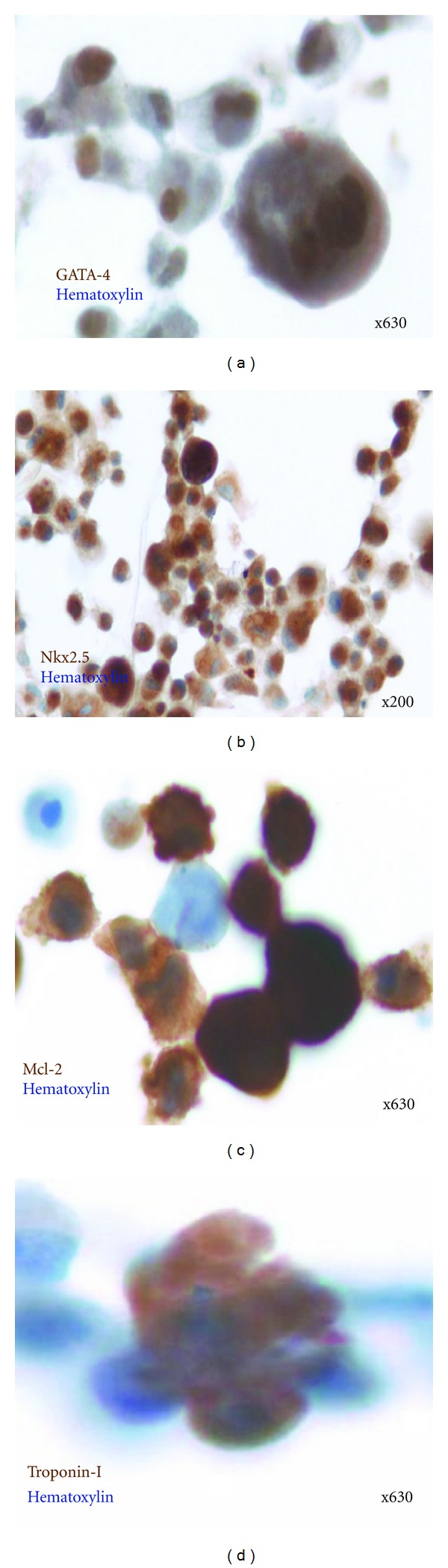
*In vitro* differentiation of hc-kit^positive^ cells. Representative photomicrographs of emigrated and immune-selected hc-kit^positive^ cells demonstrating expression of mesodermal markers (GATA 4 and Nkx 2.5) and cardiac differentiation (Mcl-2 and troponin-I). Monoclonal antibody binding was visualized using DAB reporter chromogen (brown) with nuclear hematoxylin counterstain (blue). (a) GATA 4^positive^ hc-kit^positive^ cells. Magnification ×630. (b) Nkx 2.5^positive^ hc-kit^positive^ cells. Magnification ×200. (c) Mcl-2^positive^ hc-kit^positive^ cells at Day 28. Magnification ×630. (c) Troponin-I^positive^ hc-kit^positive^ cells at Day 28. Magnification ×630. Note: hc-kit^positive^ cells are positive for the four epitopes.

Between Days 14 and 28 of SEC, c-kit^positive^ cells expressed transcription factors GATA-4 (Figure 4(a)) and Nkx 2.5 (Figure 4(b)) that are expressed in mesoderm progenitor cells [28]. After Day 28 cardiac myosin light chain (Figure 4(c)) and troponin I (Figure 4(d)) were expressed in some of the c-kit^positive^ cells that were consistent with their cardiomyocyte fate [29]. Immunoselected hc-kit^positive^ cells derived by SEC did not express the hematopoietic lineage CD45 and CD34. Immunoselected hc-kit^positive^ cells derived by SEC were also predominantly negative for Sca-1 and Isl-1 whereas some of the cells derived from cardiospheres did express Sca-1, and Isl-1 (not shown).

In basic culture media supplemented with 20% FCS, emigrated hc-kit^positive^ cells would *only* proliferate and survive *in vitro* if cocultured with their atrial tissue. With SEC, the net number of cells that spontaneously emigrated from tissue and proliferated increased over time and peaked between Day 14 and 21, (Table 1) actually doubling in number. The net numbers of immunoselected hc-kit^positive^ cells at Days 7, 14, 21 and 28 were estimated in Table 1 for triplicate determinations from tissues of *N* = 3 patients with chronic heart failure. Notably, at 21 days, hc-kit^positive^ cells represented approximately 5% of emigrated cells, or approximately 240,000 cell/g tissue. (Table 1). 

**Figure 5:**
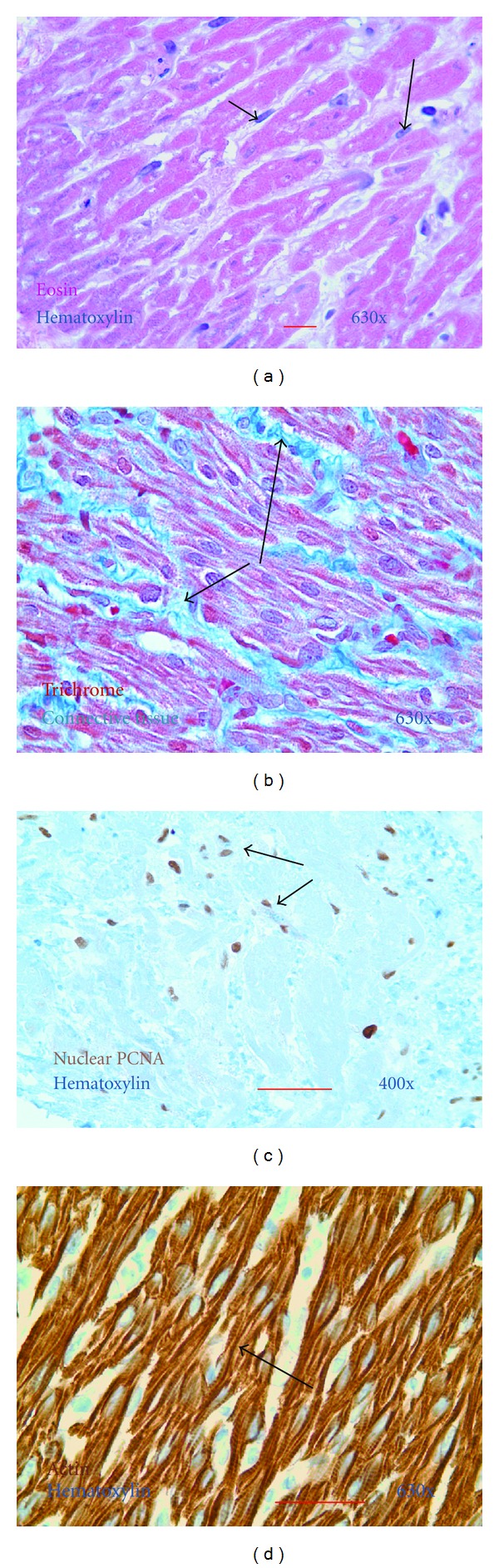
Characterization of cardiac tissue after SEC. Representative photomicrographs of tissue sections of formalin-fixed paraffin-embedded atrial tissue. The atrial appendage from a patient with end-stage heart failure was removed at the time of heart transplantation and cultured for 28 days using the SEC method. (a) Day 28 tissue explant stained with hematoxylin (blue) and eosin (red). Note preserved cardiomyocyte morphology and small nucleated interstitial cells (arrows). Magnification ×630. (b) Day 28 tissue explant stained with Trichrome. Note, a modest increase in interstitial connective tissue (blue; arrows). Magnification ×630. (c) Day 28 tissue explant immunostained to demonstrate proliferating interstitial nucleated cells. Proliferating cell nuclear antigen/PCNA^positive^ cells are brown (DAB) with hematoxylin counterstain (blue). Magnification ×400. (d) Day 28 tissue explant immunostained for muscle-specific actin are brown (DAB) and hematoxylin counterstain (blue). Note conservation of the myocellular contractile elements with visible cross-striations (arrow). Magnification ×630.

With SEC, human cardiac tissues from patients with heart failure could be maintained for extended time periods. Remarkably, Day 28 cultured atrial tissues retained fairly normal microscopic appearance (Figure 5(a)) and the actin^positive^ cardiomyocytes retained striated contractile elements (Figure 5(d)). A small increase in interstitial connective tissue was noted by Trichrome staining (Figure 5(b)). Proliferating interstitial mononuclear cells were noted by immunohistochemical staining for nuclear PCNA (Figure 5(c)).

## 4. Discussion

C-kit^positive^ cells can be derived from the right atrial appendage of children with chronic end-stage heart failure. C-kit^positive^ cells are extremely rare in normal human myocardium and the actual number is controversial. Estimates of c-kit^positive^ cells range from as low as 0.0005% of cells (~100 cells/g) in adult hearts [2] to 0.01% of cells in infant hearts [22, 30]. By confocal microscopy of sections from the right atrium of pediatric patients, c-kit^positive^ cells averaged between 3.2 and 8.9% of cells including cardiomyocytes [18]. Based on the evidence presented herein, SEC of atrial appendages from the heart in children with chronic end-stage heart failure results in the isolation of ~240,000 single hc-kit^positive^ cells/g. With SEC, ~5% of all derived cells were c-kit^positive^ at Day 21 (Table 1). This significant enrichment though is less than reported by Smith et al. [23] using a more labor intensive modification of the Messina et al. method. After selection of loosely adherent cells and cardiospheres followed by two passages, slightly less than 20% of cells were c-kit^positive^ [23].

Multiple factors could contribute to this enrichment of hc-kit^positive^ cells by SEC. With SEC the tissue does not settle onto a flask surface which, in our experience as well as others, reduces rapid fibroblast emigration from tissue leading to fibroblast overgrowth of the culture. We speculate that selection by tissue migration and emigration could contribute to this apparent enrichment of  “first responders” that, as shown herein, includes CPCs (Figure 1) presumably mobilized from niches in atrial tissue [31]. The enrichment of hc-kit^positive^ cells noted with SEC could also be due to increased *in vitro* survival and/or proliferation. One limitation of the cell counting experiments is that the number of immunoselected c-kit^positive^ cells reported in Table 1 could have included CD45^positive^c-kit^positive^ cells endothelial progenitor cells [32] or mast cells [33]. However during their characterization, the large majority of the c-kit^positive^ cells examined were noted to be CD45^neg^, and thus likely cardiac-derived stem or progenitor cells [18, 34].

With SEC, the timing of hc-kit^positive^ cell emigration from atrial tissue trended to be longer over the range of pediatric patient ages (Figure 2). In a comprehensive study, Mishra et al. described a reduced percentage of hc-kit^positive^ cells as a function of age [18]. It is possible that there will be similar age differences in the yield of hc-kit^positive^ cells using SEC with additional patient numbers at different ages.

The derivation of hc-kit^positive^ CPCs by SEC includes substantive modifications of the method originally described by Messina et al. [24] and now used widely [35]. A list of the modifications with SEC is as follows: tissues were not washed with Ca^++^-Mg^++^-free phosphate-buffered solution (PBS); tissues were not digested three times for 5 minutes with 0.2% trypsin and 0.1% collagenase IV; Dulbecco's Modified Essential Medium (DMEM) was used in place of Iscove's Modified Dulbecco's Media; DMEM was not supplemented with 2 mmol/L L-glutamine, 0.1 mmol/L 2-mercaptoethanol, thrombin, bFGF, EGF, and cardiotrophin; and, additional media was added for SEC so that the tissue explants were suspended specifically avoiding contact with culture flask surfaces. By suspending the tissue, contact-dependent fibroblast outgrowth is minimized. Without enzymatic digestion, the addition of exogenous enzymes is avoided. SEC eliminates enzymatic digestion of the tissue explants that might affect cell characterizations reliant on immunoreactive surface markers.

We have previously shown in the mouse model that removal of the cardiac tissue prevented *in vitro* proliferation of emigrated c-kit^positive^ cells under the same culture conditions used herein [21]. The addition of unfractionated bone marrow also supported the *in vitro* proliferation of emigrated c-kit^positive^ cells, removing the confounding possibility that the “supportive effect” of the cardiac tissue was to provide a continued supply of newly emigrated cells. We have preliminarily reported that emigrated hc-kit^positive^ cells in basic media supplemented with 20% FCS would only proliferate and survive to Day 28 *in vitro* if cocultured with their cardiac tissue [36]. From a technical standpoint, derivation of hc-kit^positive^ cells for patient administration without the introduction of allogeneic/xenogeneic protein prior to patient administration improves and enables translation to future clinical trials from a regulatory and patient safety perspective. C-kit^positive^ CPCs, as well as bone-marrow-derived stem cells, are believed to promote *in vivo* proliferation of resident cells and/or differentiate into cardiomyocytes by paracrine effect [18, 34]. Given the remarkable tissue preservation noted for atrial appendage following SEC (Figure 5) we speculate a tissue paracrine role in the proliferation and/or survival of CPCs. In support of the potential value of SEC as a model of cardiac paracrine function, Schittini et al. reported that media conditioned by cardiac tissue promoted the cardiomyogenic differentiation of mesenchymal stem cells [37].

## 5. Conclusions

The advances reported herein include demonstration that with the technique of SEC hc-kit^positive^ cells can be derived from the failing heart in children with chronic end-stage heart failure. These migratory cells are proliferative *in vitro* and can be enriched. Moreover, hc-kit^positive^ cells can be isolated with SEC without enzymatic digestion. Since enzymatic digestion is avoided, cell characterization is less likely to be affected during cell recovery. In clinical regenerative strategies based on autologous tissue-derived CPCs, optimizing cell characterization is a crucial safety goal. SEC is permissive for time-dependent proliferation and later cardiac differentiation. In specific, we speculate that the paracrine effects of the cocultured autologous heart tissue obviate the need for supplementing with expensive “growth factors” and coated flasks which are safety concern for clinical trials. These findings suggest that, with further studies to eliminate the use of FCS, SEC could translate well to autologous stem cell-based strategies for children with end stage HF.

## Figures and Tables

**Table 1:** Numbers of immunoselected hc-kit ^positive^ cells/g from right atrial appendage tissues of 3 patients with chronic heart failure.

	Time in culture (days)			
	7	14	21	28
Tissue: CHF^positive^ (*n* = 3)				
Total cell number*	500 ± 34	4650 ± 540	5040 ± 740	4800 ± 44
C-kit^positive^ cell number*	60 + 16	120 + 16	240 + 28	200 + 32
% C-kit^positive^	13.6	2.7	5	4.4

*Mean ± sem cells/mg wet weight.
